# Radiation pneumonitis prediction after stereotactic body radiation therapy based on 3D dose distribution: dosiomics and/or deep learning-based radiomics features

**DOI:** 10.1186/s13014-022-02154-8

**Published:** 2022-11-17

**Authors:** Ying Huang, Aihui Feng, Yang Lin, Hengle Gu, Hua Chen, Hao Wang, Yan Shao, Yanhua Duan, Weihai Zhuo, Zhiyong Xu

**Affiliations:** 1 Shanghai Chest Hospital, Shanghai Jiao Tong University, Shanghai, 200030 China; 2 Department of Nuclear Science and Technology, Institute of Modern Physics, Fudan University, Shanghai, China; 3 Key Laboratory of Nuclear Physics and Ion-Beam Application (MOE), Fudan University, Shanghai, 200433 China

**Keywords:** Radiation pneumonitis prediction, 3D dose distribution, Dosiomics, Deep learning-based radiomics, Random forest

## Abstract

**Background:**

This study was designed to establish radiation pneumonitis (RP) prediction models using dosiomics and/or deep learning-based radiomics (DLR) features based on 3D dose distribution.

**Methods:**

A total of 140 patients with non-small cell lung cancer who received stereotactic body radiation therapy (SBRT) were retrospectively included in this study. These patients were randomly divided into the training (n = 112) and test (n = 28) sets. Besides, 107 dosiomics features were extracted by Pyradiomics, and 1316 DLR features were extracted by ResNet50. Feature visualization was performed based on Spearman’s correlation coefficients, and feature selection was performed based on the least absolute shrinkage and selection operator. Three different models were constructed based on random forest, including (1) a dosiomics model (a model constructed based on dosiomics features), (2) a DLR model (a model constructed based on DLR features), and (3) a hybrid model (a model constructed based on dosiomics and DLR features). Subsequently, the performance of these three models was compared with receiver operating characteristic curves. Finally, these dosiomics and DLR features were analyzed with Spearman’s correlation coefficients.

**Results:**

In the training set, the area under the curve (AUC) of the dosiomics, DLR, and hybrid models was 0.9986, 0.9992, and 0.9993, respectively; the accuracy of these three models was 0.9643, 0.9464, and 0.9642, respectively. In the test set, the AUC of these three models was 0.8462, 0.8750, and 0.9000, respectively; the accuracy of these three models was 0.8214, 0.7857, and 0.8571, respectively. The hybrid model based on dosiomics and DLR features outperformed other two models. Correlation analysis between dosiomics features and DLR features showed weak correlations. The dosiomics features that correlated DLR features with the Spearman’s rho |*ρ*| ≥ 0.8 were all first-order features.

**Conclusion:**

The hybrid features based on dosiomics and DLR features from 3D dose distribution could improve the performance of RP prediction after SBRT.

## Background

Toxicity assessment is a very important step in radiotherapy. Radiation pneumonitis (RP) is the main complication of stereotactic body radiation therapy (SBRT) in patients with lung cancer. As per some studies, the incidence of RP ranges from 9 to 49% [1–5]. For the fact that patients treated with SBRT are prone to a fragile condition, RP may impair their quality of life and subsequently increase hospitalization and mortality rates [6–8]. Therefore, it is necessary to establish a model for predicting RP during the initial evaluation and therapeutic regimens.

Recently, the advancement in machine learning (ML) and radiomics has provided new methods for RP prediction. Quantitative medical imaging features can be extracted for computed tomography (CT) images to predict RP [9–11]. Kraf et al. proposed a predictive model for RP toxicity using pretreatment CT-based radiomics features extracted from the whole-lung volume [9], Kawahara et al. [10] and Hirose et al. [11] used radiomics features from dosimetric-based segmentation to predict the occurrence of RP. However, the occurrence of RP is affected by radiation dose, all the above prediction models for RP using radiomics features on pretreatment planning CT images rather than dose distribution. Some researchers have established RP prediction models based on some dose volume histogram (DVH) parameters, such as V5, V10, and mean lung dose (MLD) of the radiotherapy plan [12, 13]. However, it can only summarize the two-dimensional dose distribution in the target from DVH parameters, and the spatial dose distribution or organ architecture cannot be obtained from DVH parameters [14]. RP can be clinically controlled by limiting the dose to the lungs. However, dose limitation does not always prevent serious toxicities in some patients. It has been demonstrated in some studies that the voxel dose is related to the risk of tumor response, lung injury and other complications [15], and hence features extracted from the dose distribution may be of predictive significance. Thus, radiomics based on 3D dose distribution has become a more effective way to explore the toxicity induced by the radiation dose [16].

In some studies, dose-based radiomics based on 3D dose distribution is also known as dosiomics features [17–21]. Liang [20] and Adachi [21] extracted dosiomics from 3D dose distribution for RP prediction. These models for predicting RP expand the application of ML in the field of radiotherapy and promote the development of RP prediction. To the best of our knowledge, RP after SBRT has not been predicted by DLR features based on 3D dose distribution.

In this study, dosiomics and DLR features were extracted from 3D dose distribution of normal lung patients with lung cancer, and three prediction models were constructed based on random forest, including (1) a model constructed based on dosiomics features, (2) a model constructed based on DLR features, and (3) a hybrid model constructed based on dosiomics and DLR features. Besides, the correlation between dosiomics features and DLR features from 3D dose distribution was analyzed. The establishment of an accurate prediction model for RP is expected to realize the dose increase for low-risk patients or the treatment optimization for high-risk patients. This would further minimize the incidence of RP and significantly benefit cancer patients receiving radiation therapy.

## Methods

### Study cohort

**Fig. 1:**
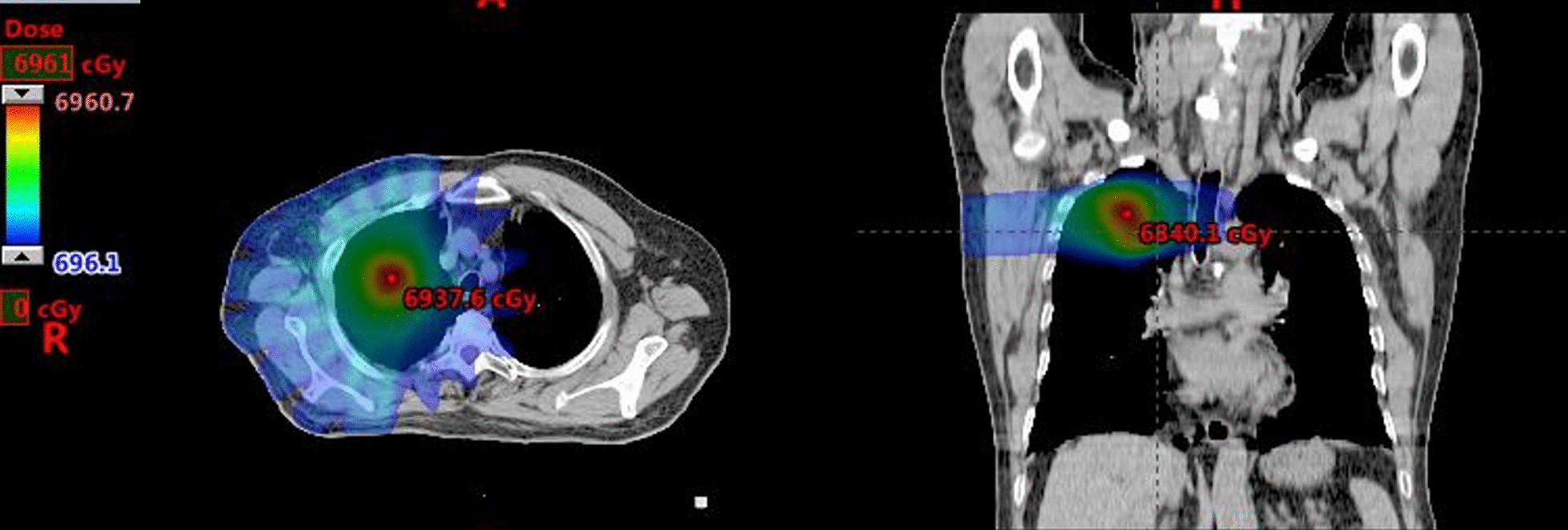
An example image of a dose distribution

A total of 140 patients who were admitted to our hospital from 2019 to 2021 were included for retrospective analysis. All patients provided written informed consent before enrollment. Patients were performed with 4-dimensional computed tomography (4D-CT). The gross tumor volume (GTV) was delineated on ten respiratory phase-sorted 4D-CT datasets. The internal target volume (ITV) was generated by performing the union of the 10-phase sorted GTVs. All patients were treated using an ITV-based strategy with an additional ITV-to-planning target volume (PTV) margin of 5 mm. The entire lung, excluding the ITV (Lung-ITV), was regarded as a normal lung. The dose distribution was calculated by Collapsed cone Convolution Superposition (CCCs) algorithm on the Pinnacle treatment planning system (TPS), with the grid size being 2.5 mm × 2.5 mm× 2.5 mm. An example image of a dose distribution was shown in Fig. 1. The patients were treated with 6 MV X-rays; the prescribed dose was 50 or 60 Gy in 4–8 fractions at an isocenter, with 95% volume of PTV was covered by the prescription dose.

**Fig. 2:**
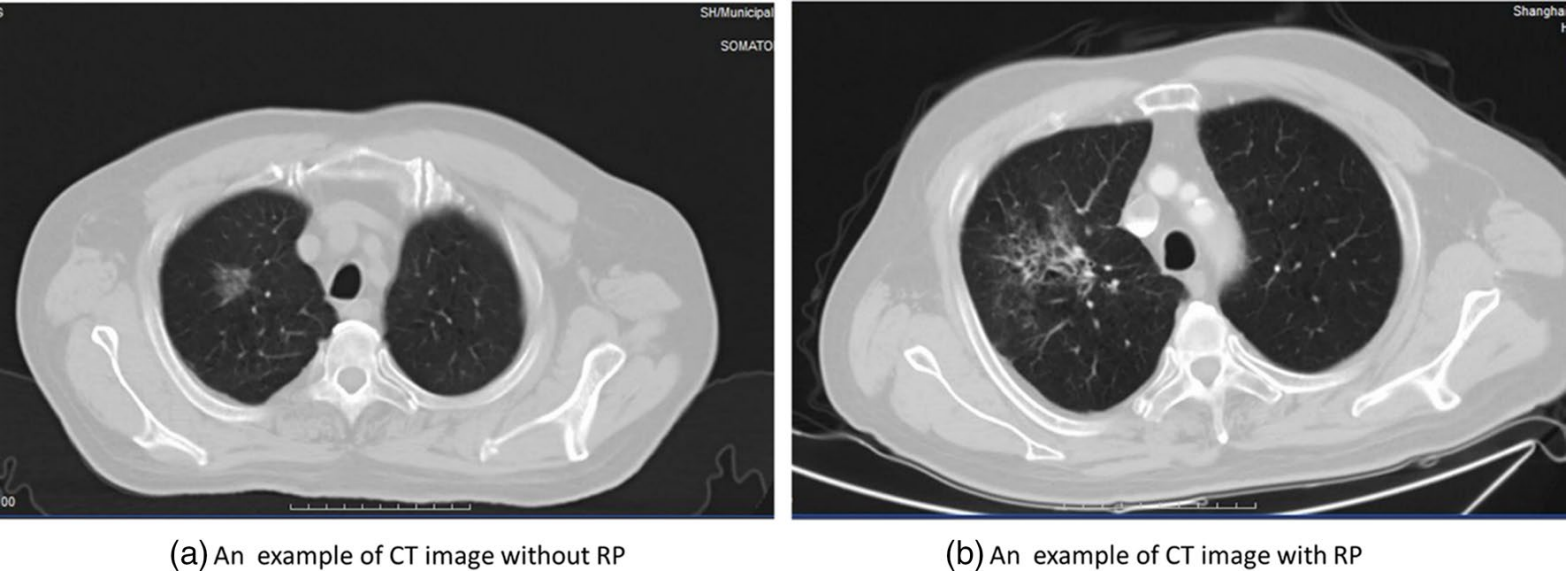
CT image examples of a CT without/with RP

**Fig. 3:**
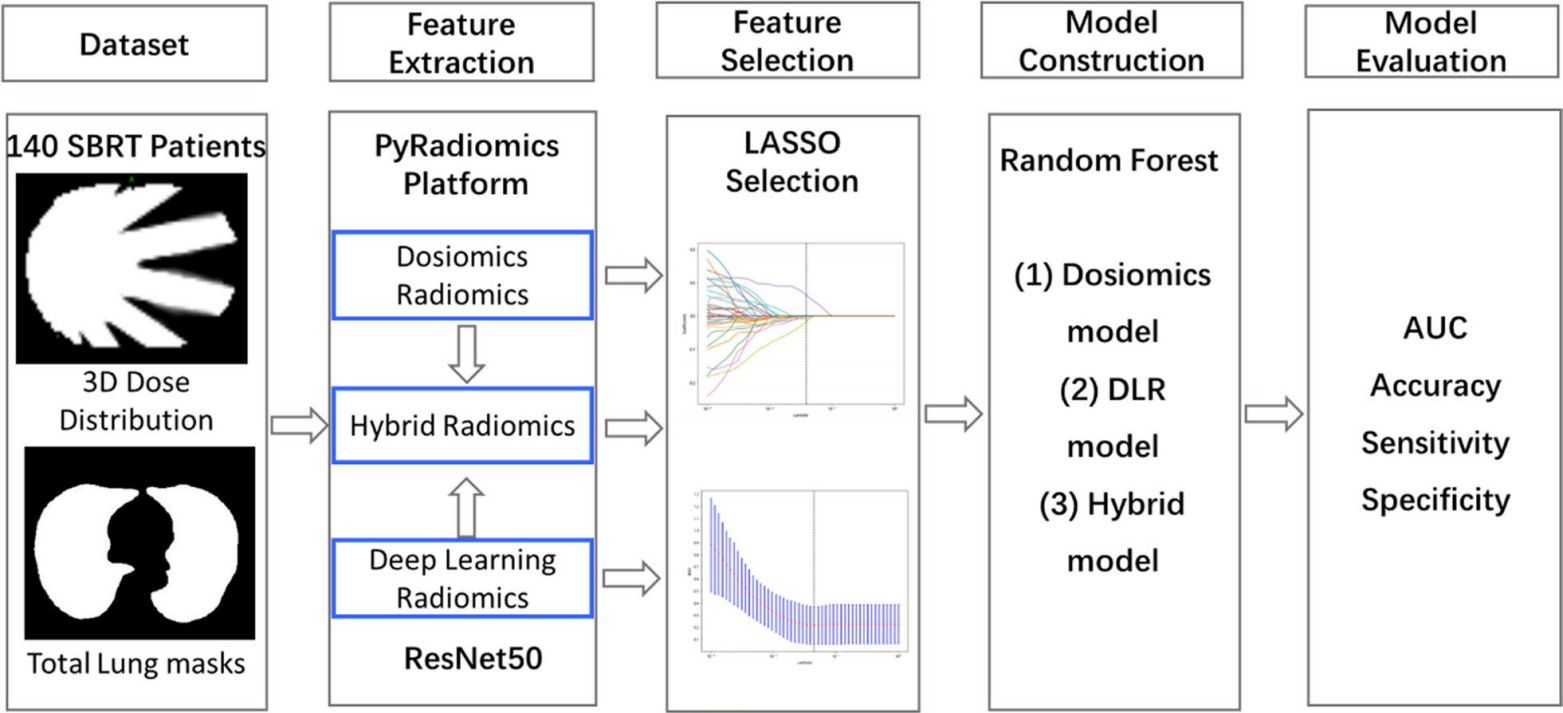
Overall workflow of RP prediction performed in this study

Patients were followed up every month after treatment completion until 6 months, and every 3 months thereafter. Each patient was performed by chest X-ray or CT at each follow-up visit. During routine follow-up, the cases were evaluated in terms of RP based on clinical findings (e.g., dyspnea, cough, pain, and low-grade fever) and radiological findings. Once diagnosed, RP was further graded by at least two radiation oncologists according to the Common Toxicity Criteria for Adverse Events (CTCAE) version 5.0 [22]. Grade 1: RP with symptoms or radiographic features without the need for steroids; Grade 2: RP requiring steroids or with symptoms that interfered with daily activities; Grade 3: RP requiring oxygen and steroids; Grade 4: RP requiring intubation. A diagnosis of RP grade ≥ 2 was defined as the primary end point. CT image examples of a CT without/with radiation pneumonitis were shown in Fig. 2. Patients with Grade 2 or higher (Grade ≥ 2) were labeled as having developed RP. A total of 40 patients were assessed as having RP with Grade ≥ 2. These 140 patients were randomly divided into the training set (n = 112, including 34 RP cases) and the test set (n = 28, including 6 RP cases). The design flow of this study is shown in Fig. 3.

### Dosiomics and DLR feature extraction

The dosiomics features were extracted automatically by Pyradiomics (https://pyradiomics.readthedocs.io/en/latest/) [23], including 14 shape features, 18 first-order features, and 75 texture features. ResNet-50 architecture was adopted to develop the deep convolutional neural networks for DLR feature extraction [24]. The 3D distribution images were cropped and resized to 96*96*96.

### Feature selection

First, redundant features were eliminated through Spearman’s correlation coefficient (CC) analysis. Normalization may reflect the difference of prescribed dose. Here, as there was no significant difference in prescribed dose between the RP and non-RP groups, the normalized z-score was used for feature selection and RP classification in this study. Subsequently, Spearman’s CCs were calculated. One of the two features that were highly correlated with the other remaining features would be eliminated if the CC between two kinds of features was ≥ 0.9 [25]. Next, least absolute shrinkage and selection operator (LASSO) [26] was employed to select a subset of features with predictive significance for each of the three binary classification models.

### Model construction and performance

A random forest model was selected as the classifier that was widely used in radiomics and achieved good performance in many studies [27]. The area under the curve (AUC) score was used to test the performance of the prediction model. The optimal cut-off value by Youden index was calculated in the process of model construction and integrated into the calculation of the accuracy, sensitivity, and specificity.

### Dosiomics and DLR feature correlation

In this study, we correlated the dosiomics features and DLR features through Spearman’s rank CCs. Besides, the correlation analysis was visualized by the Circos software (http://circos.ca)[28]. The feature sets with a correlation coefficient larger than 0.8 were selected to avoid over-cluttering during visualization.

### Statistical analysis

The Spearman’s correlation, LASSO regression, random forest classifier, and ROC curve analysis (evaluating the performance of binary classifiers) were conducted by the “sklearn” package, and the DLR features were extracted by the “PyTorch” package. The differences in clinical characteristics between patients with RP and without RP were evaluated by the t-test and Chi-square test. *P* value < 0.05 was considered statistically significant.

## Results

### Plan and clinical characteristics

A total of 140 patients (102 males and 38 females; median age: 65.5) were included in this study, including 40 RP patients (27 males and 13 females; median age: 67) Grade ≥ 2. There was no significant difference in age, gender, tumor location, ITV volume, dose fractionations, V5, V10, V20, and MLD between RP and Non-RP. The plan and clinical characteristics of these patients are listed in Table 1.

**Table 1:** Patient clinical and treatment characteristics

Clinical and treatment characters	RP	Non-RP	*P*
Age (years)	67 (33–84)	65 (47–85)	0.141
Sex			0.367
Male	27	75	
Female	13	23	
Tumor location			0.165
Left	16	53	
Right	24	47	
ITV Volume (cc)	9.84 ± 9.00	10.89 ± 12.02	0.411
Dose fractionations			0.626
60 Gy/8 fractions	12	31	
50 Gy/4 fractions	6	18	
50 Gy/5fractions	21	48	
Others	1	3	
Volume dose			
V5	18.02 ± 7.54	15.27 ± 6.64	0.221
V10	11.08 ± 4.70	8.74 ± 3.95	0.250
V20	5.49 ± 2.85	4.34 ± 2.53	0.547
MLD (Gy)	3.39 ± 1.54	3.28 ± 1.41	0.394

### Model performance

There were 22, 10 and 12 features in the dosiomics model, DLR model and hybrid model, respectively. The optimal cut-off value of the dosiomics, DLR, and hybrid models was 0.60, 040, and 0.50, respectively, in the training set, while that of dosiomics, DLR, and hybrid models in the test set was 0.60, 0.40, and 0.60, respectively.

**Fig. 4:**
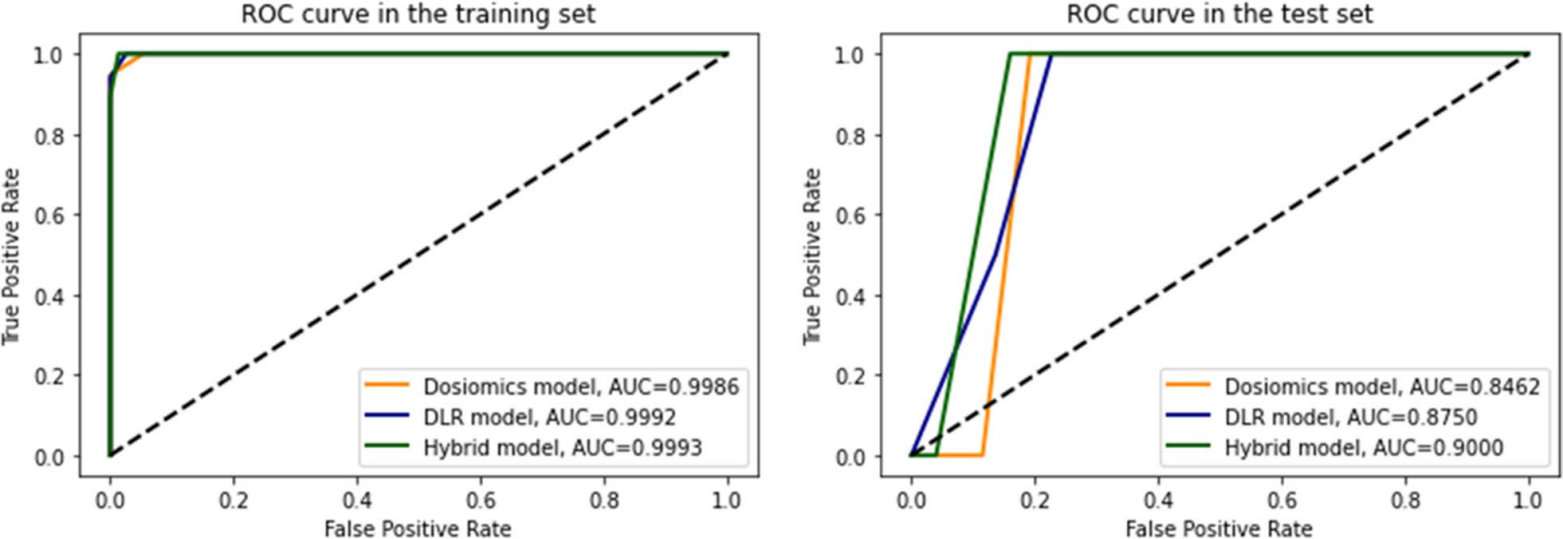
AUCs of three models in the training set and test set

The ROC curve of different models in the training and test sets are presented in Fig. 4. The AUC of three models was larger than 0.99 in the training set; While, the AUC of the dosiomics, DLR, and hybrid models was 0.8462, 0.8750, and 0.900, respectively, in the test set. The accuracy, AUC, sensitivity, and specificity of dosiomics, DLR, and hybrid models in the training and test sets are listed in Table 2. The accuracy of dosiomics, DLR, and hybrid models in the training set was 0.9643, 0.9464, and 0.9642, respectively; While that of dosiomics, DLR, and hybrid models in the test set was 0.8214, 0.7857, and 0.8571, respectively. This indicated that combining dosiomics and DLR features could improve the model performance of RP prediction.

**Table 2:** Performance metrics of three models

	Accuracy	AUC (95%CI)	Sensitivity	Specificity
Dosiomics model				
Training	0.9643	0.9986 (0.9962–1.000)	0.9474	1.000
Test	0.8214	0.8462 (0.7156–0.9767)	1.000	0.9130
DLR model				
Training	0.9464	0.9992 (0.9978–1.000)	1.000	0.9744
Test	0.7857	0.8750 (0.7508–0.9992)	1.000	0.8095
Hybrid model				
Training	0.9642	0.9992 (0.9977–1.000)	1.000	0.9867
Test	0.8571	0.9000 (0.7895–1.000)	1.000	0.8750

### Correlation between dosiomics features and DLR features

The results obtained from correlation analysis based on the Spearman’s correlation (represented by ρ) are listed in Table 3. For a quantification purpose, the number of ρ with an absolute value > 0.8 was counted. Group A was the Spearman’s rho |*ρ*| ≥ 0.8 between dosiomics features and DLR features. Group B was the Spearman’s rho 0.5 ≤ |*ρ*| < 0.8 between dosiomics features and DLR features. Besides, the ratio of the number of correlated feature pairs to the total number of feature pairs was calculated. The results showed that the ratio in Groups A and B was 0.02% and 4.72%, respectively, which was relatively low in both groups. In Group A with the Spearman’s rho |*ρ*| ≥ 0.8, the ratio of the number of dosiomics features correlated with DLR features to the total number of radiomics features was higher than the ratio of the number of DLR features correlated with dosiomics features to the total number of radiomics features. In Group B with the Spearman’s rho 0.5 ≤ |*ρ*| < 0.8, an opposite result was obtained.

**Table 3:** Correlation analyses between the dosiomics and DLR features using the Spearman’s rho

Setting^a^	No. of features	Total feature pairs	Correlated pairs and features^b^	Ratio of correlations (%)^c^
A	107:1316	140,812	(25, 6, 19)	(0.02, 5.61, 1.44)
B			(6645, 54, 736)	(4.72, 50.47, 55.93)

^a^Setting A: the Spearman’s rho |*ρ*| ≥ 0.8 between the dosiomics and DLR features and B: the Spearman’s rho 0.5 ≤ |*ρ*| < 0.8 between the dosiomics and DLR features

^b^Format (l, m, n): l is the total number of feature pairs that were correlated, m is the number of dosiomics features correlated with DLR features, and n is the number of DLR features correlated with dosiomics features

^c^Format (r, r_r_, r_c_): r = number of correlations /total number of feature pairs, r_r_ = number of dosiomics features correlated with DLR features/total number of radiomics features, and r_c_ = number of DLR features correlated with dosiomics features /total number of DLR features used

**Fig. 5:**
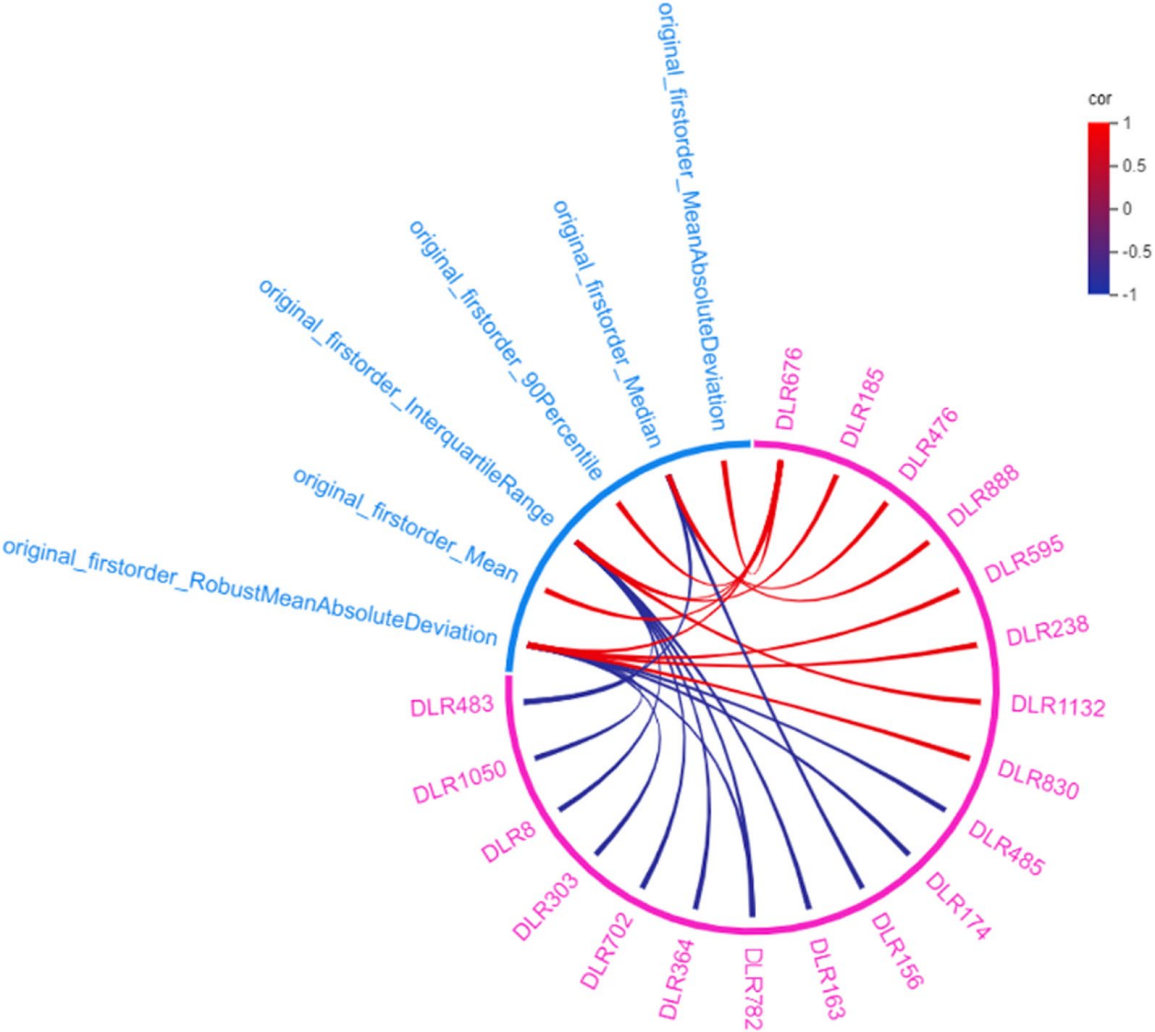
Visualization of the correlation between dosiomics features and DLR features (the Spearman’s rho |*ρ*| ≥ 0.8)

 In order to avoid over-cluttering, the correlation density in Group A was visualized by Circos (as shown in Fig. 5). The width of the link represents the correlation between the two kinds of features. The wider the link, the greater the absolute correlation. The positive correlation was represented in red color, while the negative correlation was represented in blue. All of the dosiomics features in Group A that correlated with the DLR features were identified as first-order features. The dosiomics feature with the highest correlation with DLR features was original_firstorder_InterquartileRange, original_firstorder_RobustMeanAbsoluteDeviation. The DLR feature with the highest correlation with dosiomics features was DLR 676.

## Discussions

In this study, the RP prediction model for patients with lung cancer after SBRT was established based on dosiomics features and DLR features from 3D dose distribution of normal lung. The AUC of the dosiomics, DLR, and hybrid models was 0.8462, 0.8750, and 0.900. Both the dosiomics and DLR features could be used to predict the occurrence of RP after SBRT. Importantly, combining dosiomics features and DLR features could further improve the accuracy of the prediction model. The hybrid model is feasible in clinical scenarios. The dosiomics features and DLR features can be extracted from 3D dose distribution of normal lung, and the occurrence of RP can be predicted based on the previously established model within a few minutes after the completion of the radiotherapy plan. Interestingly, the prediction model does not depend on any clinical characteristic data apart from 3D dose distribution.

SBRT is the standard therapy for NSCLC patients who cannot receive surgery and could achieve favorable clinical outcomes [29]. Given that most patients receiving SBRT have severe comorbidities or are in a vulnerable state, RP should be prevented and/or actively managed. It is necessary to predict the occurrence of RP for the reason that it may reduce the benefits of SBRT. RP is directly related to dose information. Most clinical prediction models for RP only rely on clinical factors and DVH parameters. However, DVH cannot effectively explain spatial dose distribution or organ structure. Buettner et al. proved the importance of dose distribution relative to DVH in predicting the toxicity in patients with advanced rectal cancer, and the specific information provided by 3D dose distribution can better explain the relationship between dose information and toxicity [30]. Dosiomics features are statistical, geometric, or textural measures and they can provide quantitative measurements of the intensity, shape, or heterogeneity of a given volume of interest (VOI) in medical images [31]. When applied to dose distribution, these features may be related to the inhomogeneity of dose distribution [32]. Normalization may reflect the difference of prescribed dose. Here, as there was no significant difference between the RP and non-RP groups, the normalized to z-score was used for feature selection and RP classification in this study. DLR features have been applied to disease diagnosis and prediction [33, 34]. The results of these studies have confirmed the potential of DLR features combined with dosiomics features in predicting RP.

The ML- or DL-based prediction models are highly dependent on datasets, and hence it is difficult to make a direct comparison between different studies due to different data sets. AUC can be used to compare the prediction performance of different models from different studies. For instance, the AUC of a model established by Liu et al. based on the clinical and DVH parameters was 0.76 [35]. In a study of RP prediction based on 3D dose distribution, Adachi et al. obtained an AUC of 0.837 ± 0.054 based on dosiomics features [21], which was at the same level of accuracy as the AUC of 0.846 in our study based on dosiomics features only. In this study, the DLR model outperformed the dosiomics model, and the hybrid model achieved the best performance. This indicated that combining dosiomics features and DLR features based on 3D dose distribution can improve the accuracy of RP prediction.

To the best of our knowledge, this is the first study to extract DLR features from 3D dose distribution to predict RP after SBRT. The correlation analysis was conducted between dosiomics features and DLR features. In this study, 0.8 and 0.5 were selected as the cutoff value, and there was a low correlation between dosiomics features and DLR features. There was little overlap in the RP-discriminative information expressed by these two groups of features. For the Spearman’s rho |*ρ*| ≥ 0.8, the dosiomics features that correlated with the DLR features were all identified as the first-order features. Among them, original_firstorder_Median was applied to model established. The DLR features that correlated the original_firstorder_Median with the Spearman’s rho |*ρ*| ≥ 0.8 included DLR 156, DLR676, DLR 483, and DLR 888, and they were not applied to model training. Different from dosiomics features, these DLR features have better performance in predicting RP.

However, there is a lack of an external testing cohort in this study. Nevertheless, the dosiomics and DLR features from 3D dose distributions can still be demonstrated to have benefits to RP prediction. Currently, collecting additional data from new patients represents a significant challenge, while it is an essential task for obtaining an even greater clinically relevant accuracy in predicting RP. Thus, data sharing collaboration and distributed learning suggested by Lambin et al. may play a key role in radiation oncology [36]. It is possible to establish an accurate prediction model for RP after SBRT based on sufficient multi-center data.

## Conclusion

In this study, an ML model based on dosiomics and DLR features could effectively predict RP after SBRT, which indicates that hybrid radiomics is expected to be applied to RP prediction.

## Abbreviations

RP: Radiation pneumonitis
DLR: Deep learning-based radiomics
NSCLC: Non-small cell lung cancer
SBRT: Stereotactic body radiation therapy
PTV: Planning target volume
AUC: Area under the curve
ML: Machine learning
CT: Computed tomography
DVH: Dose volume histogram
MLD: Mean lung dose
4D-CT: 4-Dimensional computed tomography
GTV: Gross tumor volume
ITV: Internal target volume
CCCs: Collapsed cone convolution superposition
TPS: Treatment planning system
CC: Correlation coefficient
SD: Standard deviation
LASSO: Least absolute shrinkage and selection operator
TP: True positive
